# Don't Get Lost in Translation: Integrating Developmental and Implementation Sciences to Accelerate Real-World Impact on Children's Development, Health, and Wellbeing

**DOI:** 10.3389/fpubh.2022.827412

**Published:** 2022-04-14

**Authors:** Lauren S. Wakschlag, Amy L. Finlay-Jones, Leigha A. MacNeill, Aaron J. Kaat, C. Hendricks Brown, Matthew M. Davis, Patricia Franklin, Cady Berkel, Sheila Krogh-Jespersen, Justin D. Smith

**Affiliations:** ^1^Department of Medical Social Sciences, Feinberg School of Medicine, Northwestern University, Chicago, IL, United States; ^2^Institute for Innovations in Developmental Sciences, Northwestern University, Chicago, IL, United States; ^3^Early Neurodevelopment and Mental Health Team, Telethon Kids Institute, Nedlands, WA, Australia; ^4^School of Population Health, Curtin University, Bentley, WA, Australia; ^5^Department of Psychiatry and Behavioral Sciences, Feinberg School of Medicine, Northwestern University, Chicago, IL, United States; ^6^Department of Preventive Medicine, Feinberg School of Medicine, Northwestern University, Chicago, IL, United States; ^7^Department of Pediatrics, Feinberg School of Medicine, Northwestern University, Chicago, IL, United States; ^8^Ann & Robert H. Lurie Children's Hospital of Chicago, Chicago, IL, United States; ^9^College of Health Solutions, Arizona State University, Tempe, AZ, United States; ^10^Department of Population Health Sciences, Spencer Fox Eccles School of Medicine at the University of Utah, Salt Lake City, UT, United States

**Keywords:** developmental science, implementation science, translation, impact, research pipeline, team science

## Abstract

Translation of developmental science discoveries is impeded by numerous barriers at different stages of the research-to-practice pipeline. Actualization of the vast potential of the developmental sciences to improve children's health and development in the real world is imperative but has not yet been fully realized. In this commentary, we argue that an integrated developmental-implementation sciences framework will result in a translational mindset essential for accelerating real world impact. We delineate key principles and methods of implementation science of salience to the developmental science audience, lay out a potential synthesis between implementation and developmental sciences, provide an illustration of the Mental Health, Earlier Partnership (MHE-P), and set actionable steps for realization. Blending these approaches along with wide-spread adoption of the translational mindset has transformative potential for population-level impact of developmental science discovery.

## Introduction

A major obstacle to improving children's health and social-emotional wellbeing is the nature of development itself, including pace, self-righting tendencies, and the formative role of environmental influences (1). This dynamic nature of development undermines decisional certainty in risk determination regarding *when to act, with whom, and how*. Further, entrenched conceptual and methodologic obstacles fuel a substantial gap between research evidence and application with the intended populations in “real-world” systems (2, 3). Traditional siloing of discovery-oriented and applied developmental sciences and the outdated notion of a sequential translational pipeline are also impediments (4). Contemporary implementation models emphasize a rapid, dynamic and iterative process including continuous evaluation of when evidence is “good enough” to implement (5, 6).

**Figure 1:**
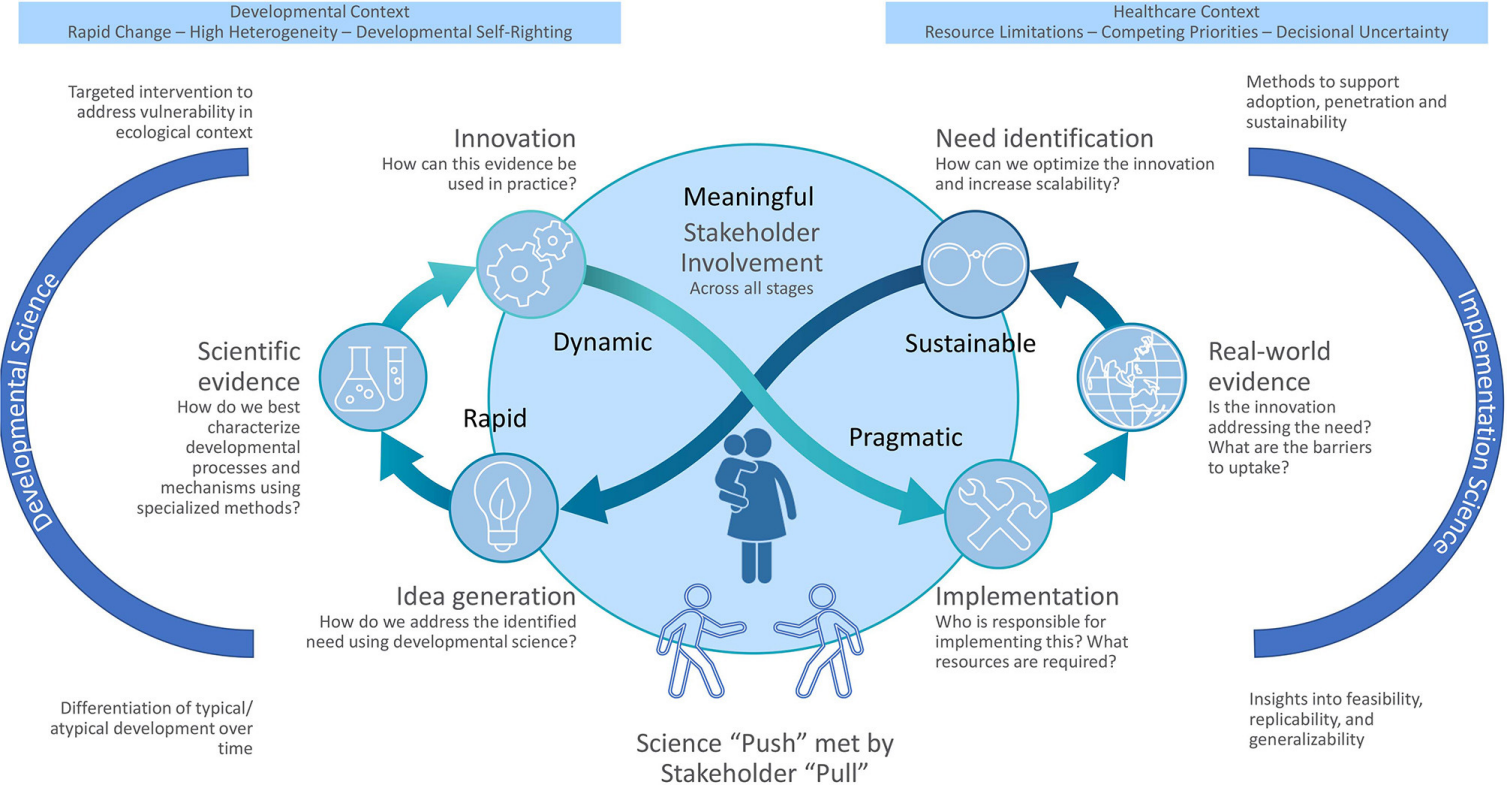
Integrated developmental-implementation sciences framework.

To advance this “science-to-impact quest” (5), we propose an actionable framework integrating developmental and implementation sciences, toward greater integration of translational thinking into the developmental sciences. Developmental science typically focuses on the individual or family, emphasizing in-depth assessments. In contrast, implementation science employs methods to promote systematic uptake, integration, and sustainment of research into real-world delivery systems implementing a policy, practice, tool, or intervention (2, 7). Although implementation science has typically focused on healthcare, it is relevant across multi-sectorial developmental contexts (e.g., childcare, and educational settings) (8, 9). Heuristically, insights from developmental science drive innovations based on characterizing developmental process in context, while implementation science guides real-world uptake of these innovations. However, this process ought not to be sequential or each aim pursued by separate teams (10). Consistent with recursive models (11), Figure 1 highlights this blended developmental-implementation framework. It is bidirectional and continuous, with innovators developing, testing, and “pushing out" discoveries and stakeholders creating “pull” for innovations aligned with needs and preferences as evidence evolves (6). We explicate core implementation science principles, illustrate with a developing initiative, the Mental Health, Earlier Partnership (MHE-P), and lay out future directions.

**Figure 2:**
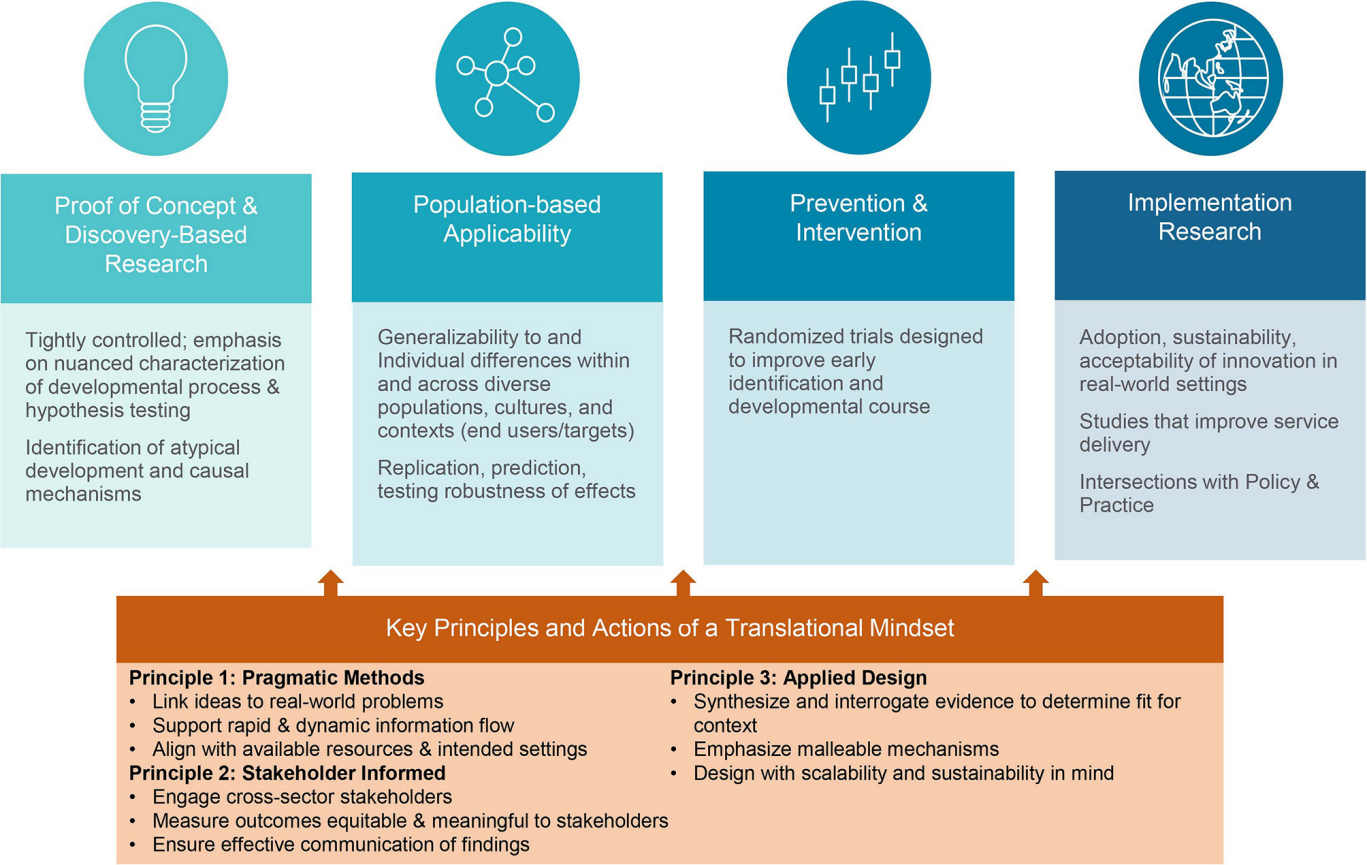
Translational mindset heuristic across the developmental sciences research spectrum.

This blended developmental-implementation sciences framework will result in a ***translational mindset*** that accelerates research-to-practice application. Translation moves discovery into programs and policies that mitigate problems in human development, health, and functioning (4). Our concept of “translational mindset” is a multi-faceted heuristic concerning how researchers *prioritize, formulate, design, enact, and disseminate* research. We emphasize *mindset* rather than action *per se* because its essence is incorporating these considerations into scientific thinking and innovation (rather than suggesting that all scientists must directly engage in applied implementation endeavors). There are a number of exemplary developmental translational efforts, largely in prevention science [e.g., (5, 12–16)]. However, these are the exception. The translational mindset underscores that for significant impact, research must be formulated with real-world problems in mind. Figure 2 highlights its salience across the developmental science translational sequence (4).

Below are core implementation science principles and their salience to developmental research:

### Principle 1: Pragmatic Methods

Pragmatic methods should be generalizable to diverse populations, feasible in real world settings, sensitive to change, publicly available, and developed and tested with efforts to eliminate or reduce potential bias (17). Pragmatic considerations occur from conception through to dissemination (10). A translational mindset requires viewing priorities, pragmatics, and feasibility outside a rarified research context (e.g., minimizing burden on respondents and systems and producing interpretable metrics). Implementation typically relies on brief or automated assessment methods because it requires ongoing data collection in resource-limited settings without specialized supports (2, 18–20). This contrasts with the typical specialized developmental science methods requiring intensive training and in-person assessment. We propose raising pragmatic concerns to an equal position with developmental considerations at the measurement selection table (21, 22). This includes considering what is “good enough” to address specific questions of interest (e.g., when is direct developmental assessment needed vs. a developmental screening questionnaire will suffice), employing modern psychometrics to improve measurement efficiency and weighting intensity of measurement burden in selection (23).

Second, pragmatics involves supporting a *dynamic and actionable information flow* between researchers and implementation systems (24). The historical separation of evidence-generators from evidence-users creates confusion regarding diffusion of responsibility. Real-world considerations are introduced from the inception including, “who may benefit?,” “how will this be used?,” and “what are uptake barriers?” Achieving a simultaneous push-pull requires iterative exchange between innovators and systems toward bi-directional “fit” (25).

### Principle 2. Stakeholder Engagement

Stakeholder engagement at every research stage ensures that aims, methods, and outputs are relevant, equitable, meaningful, and feasible for people and systems for which they are designed. The most groundbreaking discovery will not realize its potential for real-world impact if not framed in a way that matters to stakeholders. While stakeholder engagement diverges from the traditional investigator-driven, lab-based approach of developmental sciences, this reorientation is intended to catalyze the salience and impact of developmental research [for skills relevant to this reorienting see (26)]. The first key action is to *identify and engage diverse, cross-sector stakeholders*, such as *program developers, community members* (e.g., caregivers of young children), *implementing agents*/*organizations* (e.g., pediatricians, educators, and early interventionists), and *systems-level influencers* (e.g., policymakers, financers of services). Representation of diverse voices is essential to ensure equitable and meaningful approaches across contexts and groups (27). Varied stakeholder voices contribute a necessary, unique perspective in the complex processes involved in wide-scale implementation and ensuring translatability and acceptability to systems and people who engage with them.

Ongoing engagement of stakeholders with diverse lived and/or professional experiences, and from different sectors, ensures a bi-directional meaning-making process that supports uptake of research evidence. Early in the research process, engagement ensures that research findings are relevant and actionable by *measuring outcomes that are meaningful to stakeholders*. Stakeholder involvement in interpretation of these outcomes helps ensure equitable research narratives aligned with stakeholder needs and values (28). Finally, *ensuring effective communication of findings* is key to promoting adoption among diverse stakeholders and supporting engagement in the research translation process (26). This requires going beyond traditional academic outputs to accessible public communication with policy and practice reach.

### Principle 3: Applied Design

Central to the translational mindset is a systems perspective that continuously considers whether innovations are scalable and sustainable with equity across diverse real-world contexts. Thus, applied design first involves *synthesizing and interrogating evidence to determine fit for context*. Our integrated framework leverages developmental science emphasis on individual differences and contextual influences to provide a more nuanced, context-specific approach to implementation of new research evidence (5, 29, 30). Second, researchers are encouraged to *emphasize malleable risk and resilience markers and mechanisms*, preferably those with corollary evidence-based interventions, to drive impact-focused outcomes [see (12) for accounting of this translational journey]. Finally, researchers must *design with scalability and sustainability in mind*. For example, when designing a lab-based study to characterize infant cognitive development considering how tasks could ultimately be scaled up for use at a population level (e.g., remote delivery, real time coding, short forms). Implementation must be aligned with routine procedures of real-world systems, without specialized resources (31). This necessitates testing optimal implementation strategies to achieve this (2); [For research designs for testing implementation strategies see (32, 33)].

## Mental Health, Earlier Partnership

To illustrate the application of the translational mindset, we present the MHE-P, an evolving developmental science based-implementation initiative advancing our “healthier, earlier” mission (1, 34); [For exemplars of the later stages of this process see (35, 36)]. We share an early-stage project to make this translational mindset come alive, including touching on myriad anticipated challenges [for fuller explication of meeting the challenges to implementation of new innovations in pediatric healthcare, see (8, 31, 37)]. MHE-P is designed to achieve *scalable and sustainable implementation* of mental health risk identification and health promotion by establishing acceptability to clinicians and caregivers, and generalizability, adaptability, and implementation with fidelity in pediatrics (38). Implementation is to be guided by Figure 1's recursive process to identify needs, priorities, barriers, and facilitators from stakeholders in the design of the MHE-P implementation strategy. Because a key aspect of implementation is distributed leadership (moving from researcher to system-level leader driven) (39), MHE-P leadership includes community health system leaders to ensure a *sustainable* approach. Active *sustainment planning* will be key from the outset to *engage external stakeholders* and align with value-based incentives (38). MHE-P is described illustratively in the steps outlined below by applying a translational mindset to a developmental program of research (“the science of when to worry and when to act”). MHE-P activities are linked to the principles described above and the processes depicted in the center of Figure 1 and noted in italics as appropriate.

### Addressing the Need

MHE-P aims to address the challenges inherent to identification and modification of early social-emotional vulnerability presaging mental health risk within pediatric primary care. We determined pediatric primary care to be the optimal translational context to achieve shared goals because: (1) It is a nearly universal, non-stigmatized setting, increasing likelihood of population impact, and (2) monitoring and support of socioemotional development is central to routine well-child visits (40). For the MHE-P, we first engaged with community-based pediatric clinicians (e.g., pediatricians, family medicine physicians, pediatric nurse practitioners) who could help position our goals with issues of high salience to routine pediatric care (*Stakeholder Engagement)*. Challenges include mental health equity considerations, as children from racial-ethnic minorities are less likely to have access to early identification or evidence-based intervention, and more likely to suffer adverse consequences of bias or stigma (41–43). In instances with disparities in prevalence and outcomes, implementation strategies that account for factors, such as history of racial discrimination and access to health care, are essential (27). In MHE-P, we aim to increase equity in early identification of risk and referrals for evidence-based services—a documented disparity among pediatric racial/ethnic minorities (41).

### Science “Push” Met by Stakeholder “Pull” (Stakeholder Engagement; Pragmatic Methods)

The beginning of the MHE-P initiative was focused on the process of linking scientific innovations (“*Push”*) to real world problems in need of solutions (“*Pull”*). Problem specification within MHE-P started with the research team and our pediatric partners agreeing that presenting concerns about young children's social-emotional wellbeing are common (40) and systems for identification and prevention were deficient (44, 45). The absence of empirical parameters for differentiation of transient and normative behaviors from concerning behaviors impeding risk determination (1). Another *barrier to uptake* of screening tools for early identification is reluctance to screen because of uncertainty about action/resources upon problem detection (46). This quickly confirmed our hypothesis that a major *barrier* to effective early identification and prevention was decisional uncertainty (1). Although a number of screening instruments existed, they were lengthy, downward extensions of mental health concepts for older children (e.g., DSM syndromes) that pediatricians did not find particularly useful in the care of young children. Additionally, both parents and providers expressed concern about stigma associated with early identification of mental health risk (30). This pointed us to the need to engage stakeholders in framing communications regarding probabilistic risk that would be developmentally promotive and non-stigmatizing, and conveyed in a developmentally and socio-culturally valid manner (47, 48). As a result, a key goal of MHE-P is developing decision supports and a common, non-stigmatizing language.

Our recent survey of pediatric providers underscores the importance providers place on early identification coupled with significant uncertainty about determining mental health risk and corollary action in children 5 years of age and under (44) (“*pull”*). The absence of actionable “identify and act” guidance to address socioemotional concerns in young children (49) leads to missed opportunity for mitigating the enduring public, social, and economic impacts linked to neurodevelopmental vulnerability [e.g., (50)]. The real-world problem is the absence of practical tools for decision-making and a clear sightline to action impeding early identification and widespread promotion of young children's social-emotional wellbeing (*addressing needs*).

### Innovation and Need Identification (Stakeholder Engagement; Applied Design)

Fortunately, the “science of when to worry and when to act” is aligned with to the real-world problem of accurately interpreting indicators of risk in early childhood and acting in accordance (1). Thus, developmental measurement tools (51, 52) “embrace” development via operationalizing features that differentiate typical:atypical patterns of emotional dysregulation. As a broad indicator of socio-emotional risk, these measures assess irritability evident in early life that has cross-cutting predictive utility for common internalizing, externalizing, and related adaptational problems (53) (*utility of research evidence in practice*). However, we soon realized validation of these psychometrically robust tools had *no impact* on real world practice. Via extensive discussion with pediatric population health and implementation experts, it became clear that this science-to-impact quest would require adoption of a translational mindset, cross-sector collaboration, and a team science approach.

### Fit With the Context and Workflow (Stakeholder Engagement; Applied Design)

Alignment with the intended setting and resources was key. Although we had broadly aligned our scientific objectives with the expressed needs of the primary care setting, significant feasibility challenges remained, including: (a) Our irritability survey being too long for routine screening indicating a need to *optimize the innovation*; (b) the absence of decision supports (e.g., what score signifies that intervention is warranted; also *optimize the innovation*); (c) complexities of workflow integration and ensuring provider uptake as part of routine care (*implementation*) and; (d) the disconnect between the science of irritability vs. providers' and caregivers' ideas about social-emotional wellbeing in young children [e.g., the notion of using irritability to determine “probabilistic risk” at the vulnerability phase is a shift from a traditional medical mindset of the presence or absence of disorder (47); “*science push-stakeholder pull”*]; and (e) the dearth of routinely accessible prevention services for identified children and families (*need identification*). Thus, these irritability tools needed adaptation (e.g., psychometric reduction; *pragmatic*), expansion, reframing, and workflow integration based on *leveraging contextual resources* and pediatric *stakeholder input*.

The use of health information technologies for integration into the EHR and clinical workflow facilitates sustainment (54, 55) (*leveraging contextual resources*). EHR-based algorithms are important for improving equitable implementation (27) because quantification reduces reliance on social stereotyping (56). For example, the use of computer adaptive testing for precise, brief irritability screening and generation of a predictive algorithm to guide decision-making would enhance provider motivation and reduce cognitive burden (57). The goal of the algorithm is to ensure reliable and equitable personalized risk estimation through increased precision (particularly reduction of false positives in light of the rapid pace of development in early childhood). The likelihood that neurodevelopmental vulnerability—indicated by elevated irritability—will result in heightened mental health risk, is shaped by the child's unique developmental context. To account for risk amplifying and resilience promoting contextual influences, weighted risk algorithms leveraging routine information available within the EHR can then generate an individual child's risk based on consideration of developmental (e.g., language skills) and social-ecological context (e.g., ACES exposure, responsive family environment) in which behavior is embedded (23).

In this early stage, MHE-P has largely focused on barriers and strategies at the provider level [for other levels -such as, patients/families, insurers and health care providing organizations, see (58, 59)]. A significant and common implementation barrier is the gap between innovation characteristics and the realities of routine clinical care. For example, pediatricians already screen for myriad developmental and social factors. Multiple stakeholder discussions have probed whether the proposed irritability screening and predictive algorithm would link to and/or overlap with that information, adding system burden. This required a clear explication of (a) how computer adaptive irritability screening would occur automatically, (b) be integrated with the EHR, and (c) how the algorithm could reduce burden by integrating other information already collected into a single social-emotional risk score. To ensure tailoring to context, we are in the process of mapping constructs empirically demonstrated to predict mental health risk [e.g., irritability, ACES exposure (60)] to parallel or proxy data collected as part of routine care [e.g., (55)]. Framing risk estimation within non-stigmatizing, strengths-based “health” language necessitates significant input from providers and caregivers.

### Linkage to Preventive Intervention (Stakeholder Engagement; Applied Design)

While this addresses the identification challenge (i.e., when to worry), pediatric systems will require similarly scalable and sustainable evidence-based interventions (when/how to act). Further, providers are more likely to feel confident in risk identification if they have an accessible evidence-based solution to refer to. MHE-P proposes coupling early risk identification and decision support with a virtually delivered parenting intervention, the Family Check-Up 4 Health [FCU4Health; (31)]. This program has been implemented in primary care but, consistent with Figure 1, requires *adaptation* of the implementation strategy for *sustainability* (61). Thus, the FCU4Health aspect of the proposed prevention system moves from the Implementation Research domain of Figure 2 to the Prevention and Intervention domain, and back. MHE-P will develop a clinical dashboard that informs pediatric providers of families' intervention-related progress aligning with the needs of the primary care system for ongoing provision of clinically useful information. Implementation strategies will be especially geared to identifying barriers to virtual engagement for under-resourced populations (*addressing community need*) (19).

## Conclusions and Future Directions

We propose an actionable translational framework to actualize the potential of developmental sciences to generate population impact in children's health and development. We discuss how principles of implementation science can guide key actions to support developmental scientists adopting a translational mindset, with potential to heighten meaningfulness to stakeholders in child-serving systems and incorporating considerations of systems-oriented socioecological context (62). There is a key role for delivery systems and users to play in enabling, reinforcing and reimbursing evidence-driven preventive care in the developmental vulnerability phase. Meaningful framing and alignment with priorities of primary care stakeholders (caregivers of young children, pediatricians, clinic staff, insurers, health information technology experts, advocacy and policy organizations) was essential to moving the MHE-P initiative forward. Funding systems can also drive this shift to increase public health impact via creative reimagining to support the expanded duration requisite to this translational scope (63). The blending of currently disparate developmental and implementation sciences fields moves the dial toward the realization of healthier, earlier outlooks.

## Data Availability Statement

The original contributions presented in the study are included in the article/supplementary material, further inquiries can be directed to the corresponding author/s.

## Author Contributions

LW and JS conceived of the study framework. AF-J, LM, AK, CHB, MD, PF, CB, and SK-J participated in different phases of the manuscript conceptualization and refinement process. LW, JS, and AF-J collaborated in the drafting of the manuscript and its finalization. All authors have contributed to, read and approved the manuscript.

## Funding

This work was supported in part by grants MH121877 and MH107652 (PI LW), USDA 2018-68001-27550 (PIs CB and JS), DA027828 (PI CHB), and support from Northwestern University Institute for Innovations in Developmental Sciences and Telethon Kids Institute.

## Conflict of Interest

The authors declare that the research was conducted in the absence of any commercial or financial relationships that could be construed as a potential conflict of interest. The reviewer CW declared a past co-authorship with one of the authors JS to the handling editor.

## Publisher's Note

All claims expressed in this article are solely those of the authors and do not necessarily represent those of their affiliated organizations, or those of the publisher, the editors and the reviewers. Any product that may be evaluated in this article, or claim that may be made by its manufacturer, is not guaranteed or endorsed by the publisher.
